# Cytokinesis arrest and multiple centrosomes in B cell chronic lymphocytic leukaemia

**DOI:** 10.1111/jcmm.13579

**Published:** 2018-03-07

**Authors:** Marie Rogne, Oksana Svaerd, Julia Madsen‐Østerbye, Adnan Hashim, Geir E. Tjønnfjord, Judith Staerk

**Affiliations:** ^1^ Centre for Molecular Medicine Norway Nordic European Molecular Laboratory Partnership University of Oslo Oslo Norway; ^2^ Department of Haematology Oslo University Hospital Oslo Norway; ^3^ Institute of Clinical Medicine University of Oslo Oslo Norway; ^4^ Norwegian Center for Stem Cell Research Department of Immunology Oslo University Hospital Oslo Norway

**Keywords:** chronic lymphocytic leukaemia, cytokinesis, NuMA, TP53

## Abstract

Cytokinesis failure leads to the emergence of tetraploid cells and multiple centrosomes. Chronic lymphocytic leukaemia (CLL) is the most common haematological malignancy in adults and is characterized by clonal B cell expansion. Here, we show that a significant number of peripheral blood CLL cells are arrested in cytokinesis and that this event occurred after nuclear envelope reformation and before cytoplasmic abscission. mRNA expression data showed that several genes known to be crucial for cell cycle regulation, checkpoint and centromere function, such as ING4, ING5, CDKN1A and CDK4, were significantly dysregulated in CLL samples. Our results demonstrate that CLL cells exhibit difficulties in completing mitosis, which is different from but may, at least in part, explain the previously reported accumulation of CLL cells in G0/1.

## 1 INTRODUCTION

Chronic lymphocytic leukaemia (CLL) is characterized by clonal expansion of mature CD5+CD19+CD23+ B lymphocytes that accumulate in the bone marrow and lymphoid tissue, such as spleen and lymph nodes. Initially, CLL was considered to result from the accumulation of long‐lived, but resting lymphocytes; however, recent evidence points to the presence of a pool of proliferating B cells.^1^ Clinically, CLL is a very heterogeneous disease; many patients require no therapy and show an asymptomatic disease, while other patients suffer from a rapidly progressing disease despite treatment. Although therapy has been improved and has led to prolonged survival, CLL remains incurable and many patients eventually relapse.^2^

Chronic lymphocytic leukaemia is characterized by copy number variation and deletion events affecting chromosomes 6q, 11q, 13q and 17p. Chromosomal deletions are used as prognostic markers with the loss of the long arm of chromosome 13 (13q‐) being the most common aberration indicating good prognosis. Patients harbouring the 13q deletion usually live for many years without the need for therapy, while patients with a deletion of the short arm of chromosome 17 (17p‐) often require treatment and have a particularly poor prognosis and overall shortest survival of all CLL patients.^3^, ^4^

Cytokinesis is the final step of cell division ending with the physical separation into 2 daughter cells. It starts after chromosome segregation in anaphase during mitosis, with the cleavage furrow formation at the equatorial cortex ingressing inwards to divide the mother cell, and ending with the physical detachment of the 2 daughter cells. This process involves a series of spatio‐temporal regulated events ensuring an equal distribution of genomic and cytoplasmic material between the 2 nascent daughter cells.^5^

Cytokinesis failure leads to the emergence of tetraploid cells and multiple centrosomes,^6^, ^7^ although the underlying mechanism of this phenomenon remains incompletely understood. It is thought that numerical aberrations (aneuploidy) that are frequently found in tetraploid cells are connected to the presence of multiple centrosomes that block spindle geometry and, as a result, interfere with accurate chromosome segregation.^7^

In this study, we report that a significant number of CLL cells are arrested in cytokinesis. Immunohistochemistry staining for known mitosis exit markers such as Tubulin, Actin and Polo‐like kinase 1 (Plk1) was used to demonstrate that CLL cell doublets are retained at the stage of mitotic exit with multiple centrosomes in interphase. Moreover, we found that expression levels of several genes known to regulate mitotic exit and centrosome function were significantly reduced in CLL samples. Importantly, the described cytokinesis defect is distinct from the previously reported accumulation of cells in G0/1.

## 2 MATERIALS AND METHODS

### 2.1 Isolation of CD19+ cells

Mononuclear cells (MNC) from 40 mL of peripheral blood (CLL patients) or 50 mL buffy coat (healthy donors) were separated by Ficoll‐Hypaque density gradient centrifugation as described.^8^ CD19+ B lymphocytes were positively selected using magnetic beads according to the manufacturer's instructions (Miltenyi Biotec, Germany), and beads were removed from cells using multi‐sort release agent (Miltenyi Biotec). Samples were obtained following informed consent using protocols approved by the Regional Medical and Health Research Ethics Committee of South‐East Norway.

### 2.2 Immunostaining of CD19+ cells

Cells were fixed in 3% paraformaldehyde, permeabilized with 0.1% TX100 and blocked in PBS with 0.01% saponin and 3% (essentially fatty acid free) BSA. Staining was performed using primary antibodies Lamin B1 (goat) (1:200; Santa Cruz, Texas, USA), Plk1 (Rb) (1:100; Abcam, Cambridge, UK), NuMA (Rb) (1:100; Abcam), Aurora B (Mo) (1:100; Abcam), Pericentrin (1:100; Abcam), Tubulin (Mo) (1:400; Sigma, Missouri, USA) and Actin (goat) (1:200; Santa Cruz) before being exposed to secondary Alexa fluor 488 donkey anti‐mouse (1:500) (Invitrogen, Massachusetts, USA), Alexa fluor 546 donkey anti‐rabbit (1:500) (Invitrogen) and/or Alexa fluor 488 anti‐rabbit (1:500) (Invitrogen) or Alexa fluor 546 donkey anti‐goat (1:500) antibodies. Imaging was performed using a 60×/1.4 DIC oil immersion objective on a LSM510 META confocal microscope (Zeiss, Germany).

### 2.3 Extract preparation for protein analyses

For whole cell lysates, cells were lysed for 5 minutes in RIPA buffer [50 mmol/L Tris‐HCl pH 8.0, 100 mmol/L NaCl, 1% NP‐40, 0.5% Na‐deoxycholate, 0.5% TX100, 0.5% SDS, 2 mmol/L EDTA + Protease inhibitors (Roche, Switzerland)] followed by sonication. Protein concentration was measured [BCA protein assay kit (Pierce, Massachusetts, USA)] and 5‐30 μg protein extract was run on SDS‐PAGE and immunoblotted against the indicated antibodies.

### 2.4 Immunoblot analysis

After sample preparation, proteins were separated by SDS‐PAGE gel (BioRad, Criterion gels) and blotted on PVDF membrane (45 μm, 1 hour, 100 Volt). Next, membranes were blocked in 5% fatty free milk or 3% BSA before probing with primary antibodies against Lamin B1 (1:1000, Santa Cruz), NuMA (1:500, Abcam) and TP53 (1:500, Sigma). After incubation with appropriate HRP‐conjugated secondary antibodies (1:10 000, Jackson laboratories), blots were washed and developed using Super Signal West Pico/Dura substrate (Pierce).

### 2.5 RNA isolation and quantitative real‐time PCR (qRT‐PCR)

Total RNA was isolated using Qiazol and RNeasy kit (Qiagen, Manchester, UK) according to the manufacturer's instructions. SuperScript II Reverse Transcriptase (Invitrogen) and random hexamer primer were used for synthesis of complementary DNA. qRT‐PCR reactions were performed using an ABI Prism 7700 sequence detector (Applied Biosystems, Paisley, UK). SYBR green kit (Applied Biosystems) and primers specific for CDKN1A (FW 5′‐TAGCAGCGGAACAAGGAG‐3′, RW 5′‐AAACGGGAACCAGGACAC‐3′), CDK4 (FW 5′‐TTGGCAGCTGGTCACATGGT‐3′, RW 5′‐CAGATCAAGGGAGACCCTCACG‐3′), ING4 (FW 5′‐TGCGGGGATGTATTTGGAACA‐3′, RW 5′‐TTTCAGCCTTCAGGTCCTCTG‐3′), ING5 (FW 5′‐CGCCATGTACTTGGAGCACTA‐3′, RW 5′‐TTCTTATCTTCCGTCCTCTGGT‐3′), TP53I3 (FW 5′‐TTCACCAAAGGTGCTGGAGTT‐3′, RW 5′‐ACCCATCGACCATCAAGAGC‐3′) and TP53 (FW 5′‐AGGCCTTGGAACTCAAGGAT‐3′, RW 5′‐CCCTTTTTGGACTTCAGGTGF‐3′). Samples from at least 3 independent healthy donors and indicated number of CLL patient samples were used for each target gene and run in technical triplicate. Values were normalized to the reference gene hARP (FW 5′‐CGCTGCTGAACATGCTAA‐3′, RW 5′‐TGTCGAACACCTGCTGGATG‐3′).

### 2.6 RNAseq

Candidate genes involved in the described cytokinesis defect were identified based on a list of differentially expressed genes from a previously analysed, publically available RNAseq data set based on a cohort of 98 CLL patients and healthy donor controls^9^ (European Genome‐Phenome Archive, under accession number EGAS00001000374). In Ferreira et al*,* 9 genes were considered differentially expressed when showing absolute fold change (Tumour/Normal) ≥ 2 with false discovery rate (FDR) < 0.01.

For a more detailed Methods section, see Supporting Information.

### 2.7 Statistical analysis

For all statistical analyses in the manuscript, *t* test was used. Quantitative data are presented as average ± SEM, and differences with *P *≤* *.05 using *t* test were considered significant.

## 3 RESULTS

### 3.1 A significant number of CLL cells were arrested in cytokinesis

**Figure 1:**
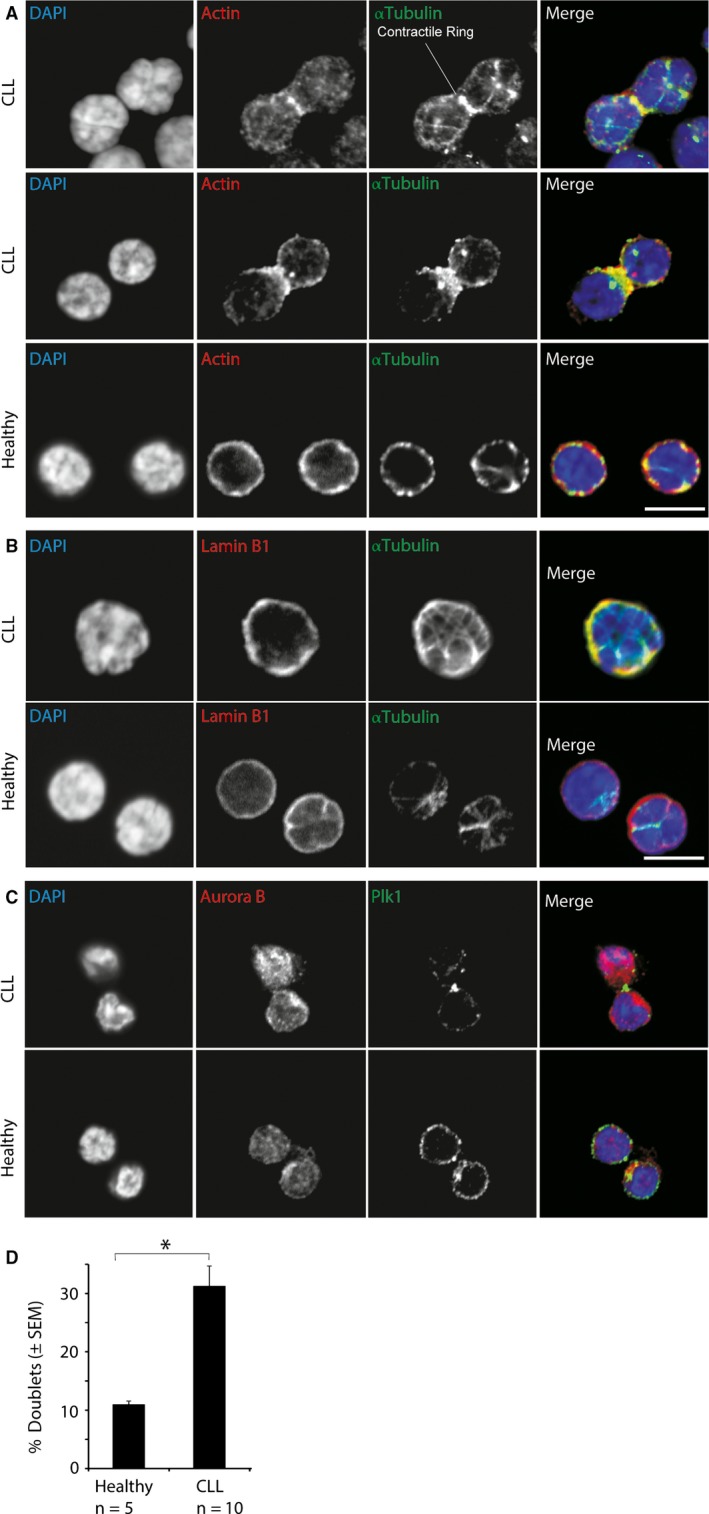
Significant number of chronic lymphocytic leukaemia (CLL) cells were arrested in cytokinesis. Representative images showing primary CLL and healthy donor CD19+ B cells stained with (A) Actin (red) and αTubulin (green) (B) Lamin B1 (red) and αTubulin (green) or (C) Plk1 (green), Aurora B (red) and DAPI (blue). D, Statistical analysis of representative images shown in (A). Number of interconnected cells was significantly up‐regulated in CLL samples (*P ≤* *.05). Approximately, 30% of CLL cells were present as doublets. CD19+ cells from CLL patients (n = 10) and healthy donors (n = 5) were counted based on αTubulin staining and connected/non‐connected cytoplasm between cells. For each donor and condition, at least 100 cells were counted. Scale bar 10 μm

Cell cycle arrest is manifested as one of the key features of CLL^10^ although the underlying molecular events remain incompletely understood. Here, we performed immunofluorescence staining for Actin and Tubulin on peripheral blood CD19+ B cells isolated from healthy donors and CLL patients (Supporting Information) and showed that approximately 30% of CLL cells were blocked at the cell cycle mitotic stage, more precisely at the step of cytokinesis (Figure 1A‐D). In contrast, we could not observe such a phenomenon in healthy donor samples (Figure 1A). Using Lamin B1 and DAPI staining, we demonstrated that the nuclear envelope was closed and chromosomes were decondensed (Figure 1B); however, in CLL doublet cells the cytoplasm was interconnected (Figure 1A). When we performed immunofluorescence staining for Plk1 and Aurora B, proteins known to regulate cytokinesis,^11^, ^12^ we found that Plk1 localized at the cytokinesis contractile ring in CLL doublets (Figure 1C), which is a hallmark of cytokinesis.^11^ Taken together, our results indicate that CLL cell doublets were in the cytokinesis stage of the cell cycle, specifically, after reformation of the nucleus, but before abscission and physical separation of the cytoplasm.^11^, ^13^, ^14^, ^15^, ^16^

**Figure 2:**
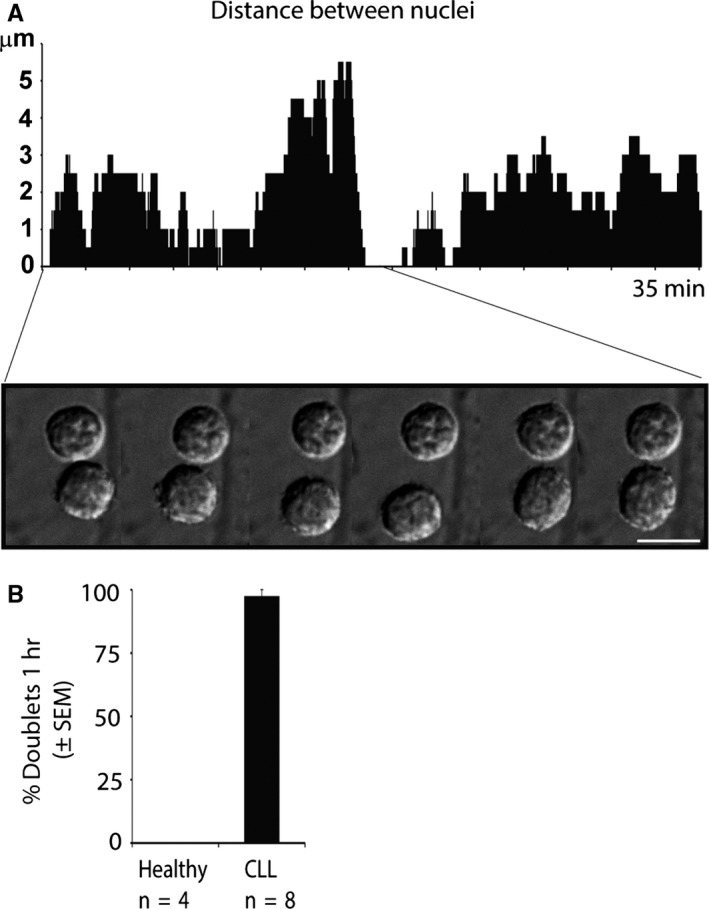
Live cell imaging of chronic lymphocytic leukaemia (CLL) cells arrested in cytokinesis. (A) Representative live cell imaging captures of freshly isolated CD19+ cells. Cells were co‐cultured on CD40L feeder cells for the entire duration of live cell imaging. Shown is the fluctuating distance between 2 nuclei of CLL doublets ranging from 0 μm to 3‐5 μm and back to 0 μm. Live cell imaging was performed on CLL (n = 8) and healthy donor (n = 4) samples with an average of 5× 1 h recording. (B) Statistical analysis of live cell imaging shown in (A). All CLL cell doublets with the exception of one were unable to separate during 1 h recording. Scale bar 10 μm

To investigate whether CLL doublets can complete cytokinesis, we co‐cultured them on CD40L feeder cells and performed live cell imaging. We observed that CLL doublets fluctuate back and forth and remained interconnected, a phenomenon we could not find in healthy donor cells (Figure 2A,B). Cell nuclei fluctuating back and forth with distances between the 2 nuclei ranging from 0 to 5 μm [CLL (Video S1) and healthy donor (Video S2)] have previously been shown to be characteristic for cells arrested in cytokinesis.^17^, ^18^

### 3.2 Cell cycle control and cytoskeletal function were dysregulated in CLL samples

**Figure 3:**
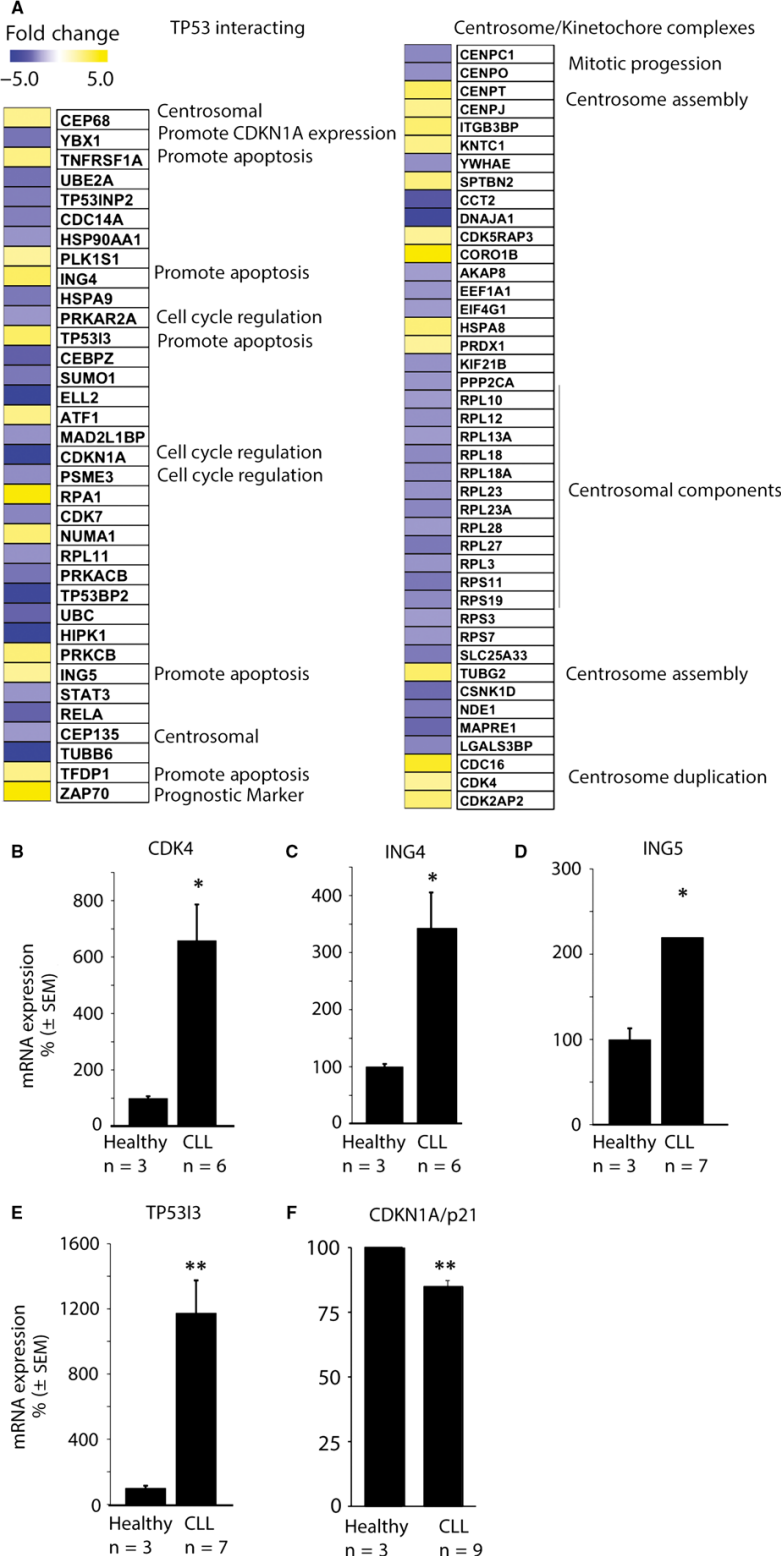
In chronic lymphocytic leukaemia (CLL) cells, cell cycle control and cytoskeletal function were dysregulated. (A) Subset of genes differently expressed in CLL cells as reported by Ferreira et al.^9^ Shown are genes involved in centrosome assembly, duplication and regulation as well as genes regulated by TP53. qRT‐PCR analyses on the indicated number of CLL vs healthy control (n = 3) samples demonstrated significant differences in mRNA expression levels for (B) CDK4 (*P ≤* *.05), (C) ING4 (*P ≤* *.05), (D) ING5 (*P ≤* *.05), (E) TP53I3 (**P ≤* *.005) and (F) CDKN1A/p21 (**P ≤* *.005)

To identify genes that possibly contribute to the observed cytokinesis arrest, we searched a list of 3578 differentially expressed genes based on a publically available RNAseq data set obtained from 98 CLL patients and healthy donor control samples.^9^ We identified several genes encoding proteins known to be involved in cell cycle regulation to be dysregulated. CDKN1A (fold change (fc = −4.94), inhibitor of growth family member 4 and 5 (ING4) (fc = 2.94) and (ING5) (fc = 2.13), tumour protein and TP53 inducible protein 3 (TP53I3) (fc = 2.92), centromere protein O (CENPO) (fc = −2.21) and proteasome activator complex subunit 3 (PSME3) (fc = −2.26)^19^, ^20^, ^21^, ^22^ were significantly altered in CLL compared to healthy donor samples (Figure 3A and Supporting Information). In addition, genes encoding proteins involved in centrosome assembly and function such as centromere protein T (CENPT) (fc = 2.94) and centromere protein J (CENPJ) (fc = 2.08) were up‐regulated in CLL compared to healthy samples (Figure 3A). Moreover, expression levels of cyclin‐dependent kinase 4 (CDK4) (fc = 2.08), cell cycle division cycle protein 16 homolog (CDC16) (fc = 3.81) and cyclin‐dependent kinase 2‐associated protein (CDK2AP) (fc = 2.69), which are involved in centrosome duplication,^23^, ^24^, ^25^ were increased in CLL compared to healthy samples (Figure 3A and Supporting Information). Next, we performed qRT‐PCR analysis to verify that genes involved in cell cycle regulation were also dysregulated in the CLL samples used to analyse the cytokinesis defect on a cellular level. Indeed, CDK4 (Figure 3B), ING4 (Figure 3C), ING5 (Figure 3D), TP53I3 (Figure 3E) and CDKN1A (Figure 3F) mRNA expression levels were significantly altered in our cohort of CLL compared to healthy donor samples. Taken together, we found several genes known to play a role in cell cycle regulation altered in CLL.

### 3.3 Basal TP53 expression levels were significantly altered in CLL samples

**Figure 4:**
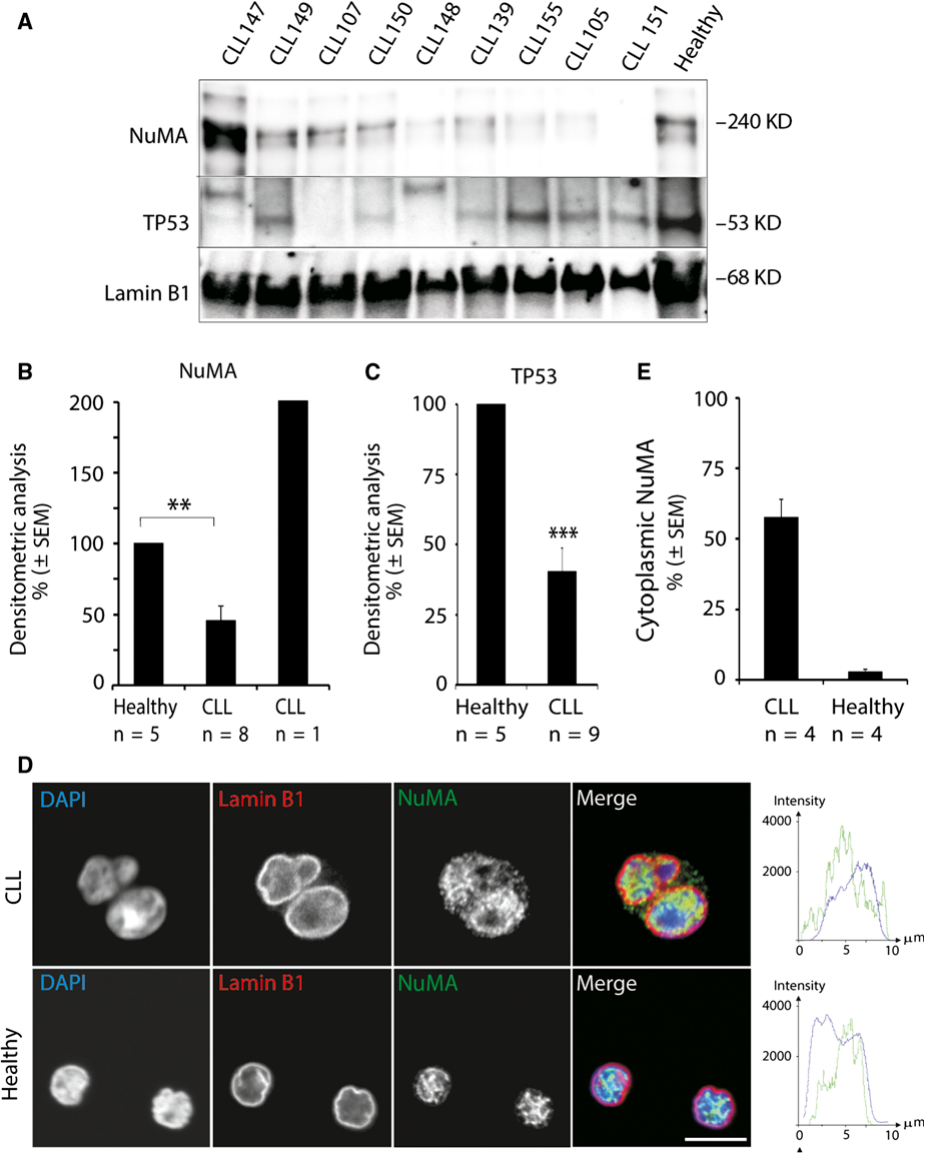
TP53 and NuMA protein levels were dysregulated in chronic lymphocytic leukaemia (CLL) samples. (A) Protein lysates of CD19+ cells (10 μg) isolated from CLL samples (n = 9) and healthy donor control were analysed for NuMA and TP53 protein levels by immunoblotting. For healthy donors, a total of 5 samples were analysed for NuMA and TP53 protein levels; however, only tone representative sample has been included in the present blot. (B,C) Densitometric analysis of NuMA and TP53 protein levels shown in (A) and normalized against Lamin B1 protein levels. Standard error of the mean (SEM) is shown. Both, NuMA (**P ≤* *.005) and TP53 (***P ≤* *.0005) protein levels were significantly decreased in CLL samples. D, Representative images of NuMA distribution in CLL (n = 4) and healthy donor (n = 4) CD19+ cells. (E) Statistical representation of NuMA staining shown in (D). At least 100 cells were counted for each donor and condition. Scale bar 10 μm

The heatmaps shown in Figure 3A contain several genes that encode proteins known to play a role in cell cycle control and to interact with TP53. As reduced TP53 levels have also been associated with accumulation of cells in cytokinesis,^25^, ^26^ we examined a potential link between TP53 dysregulation and the observed cytokinesis defect. In all analysed CLL samples, we found significantly reduced TP53 protein (Figure 4A,C) and reduced mRNA expression levels compared to healthy donor samples (Supporting Information). In one CLL sample, we could not detect any TP53, and in 2 of 9 analysed CLL samples, TP53 migrated higher, which can possibly be explained by post‐translational modifications (Figure 4A). While it has previously been shown that TP53 is dysregulated in CLL cells,^27^ to the best of our knowledge, we are the first to compare basal protein levels of TP53 between CLL and healthy donor CD19+ cells.

### 3.4 NuMA protein levels were reduced in CLL patient samples

Proteomics analysis performed on a subset of CLL samples indicated that NuMA protein expression levels might be altered in CLL (data not shown). As NuMA is known to be required for proper assembly and maintenance of the mitotic spindle, and non‐functional NuMA has been shown to cause defects in mitosis exit and inability to complete cytokinesis,^28^, ^29^ we analysed NuMA protein levels in our cohort of CLL samples. Indeed, we found a significant reduction (50%) of NuMA in 8 of 9 analysed CLL compared to 5 healthy donor samples (Figure 4A,B).

In addition, we detected cytoplasmic leakage of NuMA in CLL samples, while this was not the case in healthy donor samples (Figure 4D,E). These results further support that CLL cells exploit mitosis defects since under physiological conditions, NuMA localizes to the nucleus during interphase and is dispersed throughout the cytoplasm during mitosis.^30^, ^31^

**Figure 5:**
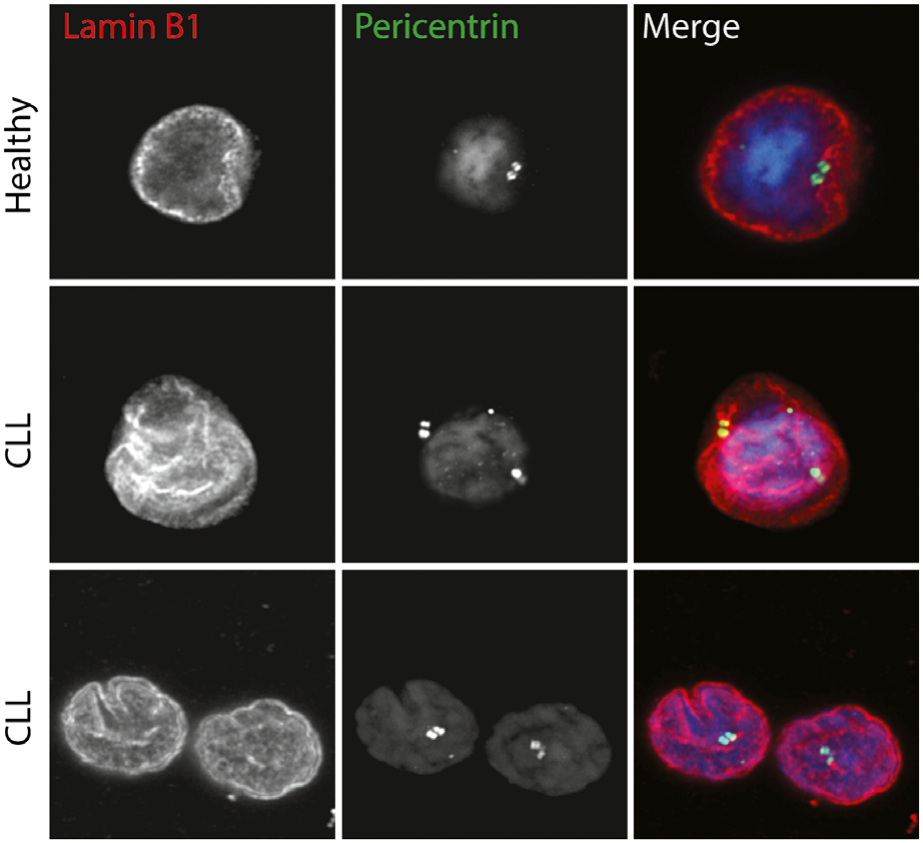
Chronic lymphocytic leukaemia (CLL) cells exhibited multiple Pericentrin‐positive centrosomes. Representative images of primary CD19+ cells stained with Lamin B1 (red), Pericentrin (green) and DAPI (blue). All analysed patient samples (n = 11) displayed multiple centrosomes, while this was not the case for any of the analysed healthy donor samples (n = 4). Scale bar 10 μm

**Figure 6:**
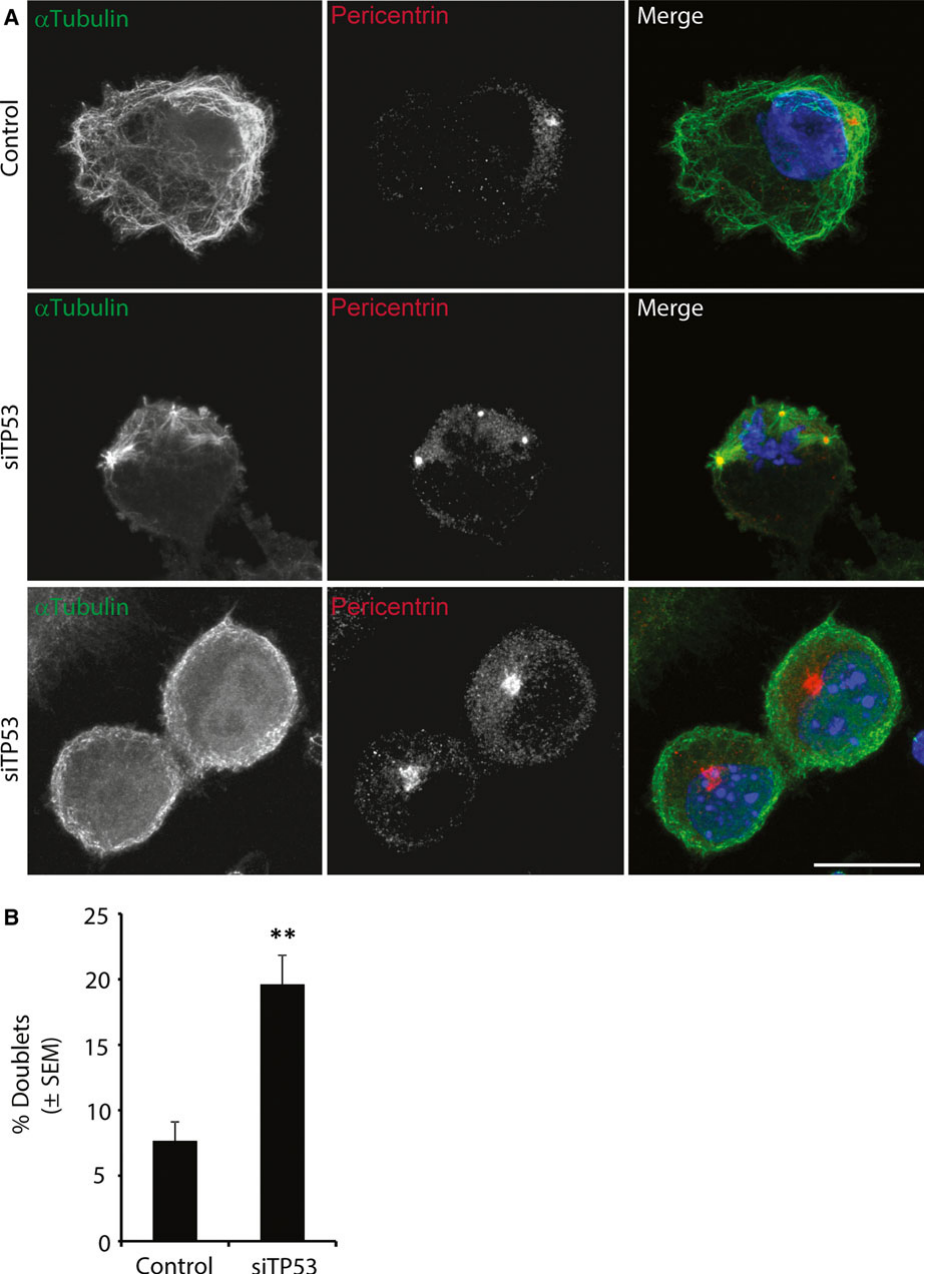
Knockdown of TP53 in healthy donor CD19+ cells led to accumulation of cells in cytokinesis and multiple centrosomes. (A) Representative images of CD19+ cells stained with αTubulin (green), Pericentrin (red) and DAPI (blue). (B) Knockdown of TP53 in healthy donor CD19+ cells led to multiple centrosomes and accumulation of cells in cytokinesis. Number of interconnected cells was significantly up‐regulated under siTP53 conditions (**P ≤* *.005). N = 4 healthy donor samples were analysed for each experimental condition, and at least 100 cells were counted for each experimental condition. Scale bar 10 μm

As reduced NuMA protein levels have also been linked to the formation of multiple centrosomes, we performed immunofluorescence staining of Pericentrin (Figure 5) and Tubulin (Figure 1) and found multiple centrosomes in all analysed CLL samples. This finding is in accordance with previous reports showing that leukaemic cancers often exhibit multiple centrosomes.^32^, ^33^ Interestingly, when we performed transient siRNAmediated knockdown of TP53 in healthy donor CD19+ cells (Figure 6 and Supporting Information), we could show that knockdown of TP53 resulted in a phenotype that highly resembled CLL cells with multiple centrosomes and accumulation of cells arrested in cytokinesis. Taken together, our results indicate that reduced NuMA and TP53 levels may contribute to the cytokinesis defects described in this study.

## 4 DISCUSSION

In the present study, we report for the first time that a significant number of CLL cells are arrested in cytokinesis. CLL cells exhibit supernumerary centrosomes and decreased expression levels of genes encoding proteins involved in cell cycle regulation and mitotic progression such as CENPO, PSME3 and CDKN1A. In contrast, genes encoding CDC16, CDK4 and CENPT, which are known to be involved in centrosome assembly and duplication, were increased in CLL samples.

The evidence that CLL cells were arrested in cytokinesis is based on Actin, Tubulin and Plk‐1 staining of a joint cytoplasmic bridge, which is distinct from the previously described overrepresentation of cells in the G0/1 cell cycle phase. The described Actin, Tubulin and Plk1 localization corresponds to late steps of cytokinesis, more precisely, to the step after nuclear envelope reformation and chromosome decondensation, but before cytoplasmic abscission by the contractile ring between the 2 daughter cells.^11^, ^13^, ^14^, ^15^, ^16^ Previous studies have linked cytokinesis defects, multiple centrosomes and accumulation of cells in G0/1 to be a consequence of dysregulation of important players in cell cycle regulation.^34^ Centrosome amplification can be induced via deregulation of the centrosome duplication process in interphase 35, 36, 37, 38, 39, 40, 41, 42, 43 or through mitotic defects resulting in G0/1 cells with tetraploid cells.^44^ The latter can be experimentally induced by overexpression of Plk1, Aurora A and Aurora B.^41^, ^44^ Our data also indicate that centrosome aberrations in malignant cells can arise via mitotic defects. The described cytokinesis failure leading to cells that harbour supernumerary centrosomes resembles the findings described for overexpression of Plk1, Evi1, Aurora A and Aurora B.^41^, ^44^

The TP53 pathway has been extensively studied in CLL, and chromosomal aberrations such as deletion of 17p13 are known to cause loss of TP53 function.^45^ In addition, decreased TP53 expression and activity independent of 17p13 deletions have been associated with deletion of the ATM gene, which increases MDM2 activity and in turn leads to decreased TP53 pathway activity 45, 46. In the present study, we detected significantly reduced basal TP53 protein levels in all analysed CLL samples, although only 2 of the 20 patients included in our study are known to harbour a TP53 mutation. Down‐regulation of TP53 is known as one cause to bypass anaphase checkpoint and DNA damage response during mitosis exit, which under normal conditions is active and prevents cells from acquiring chromosomal aberrations. Moreover, reduced TP53 levels lead to multiple centrosomes and accumulation of cells in cytokinesis.^26^, ^41^ Reduced TP53 protein levels may contribute to the cytokinesis defect described in the present study, a notion supported by our finding that siRNA‐mediated knockdown of TP53 in healthy donor CD19+ cells led to an accumulation of cells arrested in cytokinesis.

NuMA is important for maintenance of the mitotic spindle, and non‐functional NuMA has been shown to cause defects in mitosis exit and completion of cytokinesis.^28^, ^29^ These studies support our finding that reduced NuMA levels may contribute to the cytokinesis arrest described in the present study. Previous studies performing RNAi‐mediated knockdown linked reduced NuMA protein levels to reduced CDKN1A (p21) mRNA expression levels, while it does not affect expression of TP53 regulated pro‐apoptotic genes.^28^, ^29^ We also showed that CDKN1A expression levels were reduced in CLL compared to healthy donor samples, while pro‐apoptotic genes such as TP53I3, ING4 and ING5 were increased.

One key characteristic of CLL, as assessed by flow cytometry‐based analysis is the accumulation of clonal B cells arrested in the early G0/1 phase of the cell cycle.^47^, ^48^ A possible explanation why the cytokinesis defect described here has not previously been reported is that conventional flow cytometry does not easily distinguish between cells in the G1 or cytokinesis part of the cell cycle and that a majority of cells in cytokinesis are counted as G1 cells because of the break of the cytoplasmic bridge.^49^

In conclusion, our results provide novel insights into the cell cycle dysregulation that occurs in CLL. Centrosome amplification, secondary to a cytokinesis defect, might be one important contributing factor to chromosomal instability in CLL. Additionally, it may be a critical determinant for the observed growth advantage of CLL cells where TP53 is down‐regulated; once cells overcome cytokinesis and cell cycle defects, uncontrolled proliferation may be the consequence.

## AUTHOR CONTRIBUTIONS

MR, OS and JM performed experiments, AH performed bioinformatics analysis and GET provided essential research material. MR, OS, JM, AH, GET and JS analysed and interpreted data. MR, OS, JM, AH and JS wrote the manuscript. All authors read and approved the final version of the manuscript. Supplementary information is available online.

## CONFLICT OF INTEREST

The authors declare no conflict of interest.
