# Supplementary material for: Cytokinesis arrest and multiple centrosomes in B cell chronic lymphocytic leukaemia
Source: J Cell Mol Med. 2018 Mar 7;22(5):2846–55. doi: 10.1111/jcmm.13579 (PMC5908127; doi:10.1111/jcmm.13579)
Supplement: Supplementary file 1 [file JCMM-22-2846-s001.docx]

**Supplementary Information**

**Cytokinesis arrest and multiple centrosomes in B cell chronic lymphocytic leukemia**

Marie Rogne^1,#^, Oksana Svaerd^1,#^, Julia Madsen-Østerbye^1,#^, Adnan Hashim^1^, Geir E. Tjønnfjord^2,3,*^ , Judith Staerk^1,2,4, *^

*^1^Centre for Molecular Medicine Norway, Nordic European Molecular Laboratory Partnership, University of Oslo, Oslo, Norway*

*^2^Department of Haematology, Oslo University Hospital, Oslo, Norway*

*^3^Institute of Clinical Medicine, University of Oslo, Oslo, Norway*

*^4^Norwegian Center for Stem Cell Research, Department of Immunology, Oslo University Hospital, Oslo, Norway*

*#These authors contributed equally to this work*

**Suppl. Figure S1**


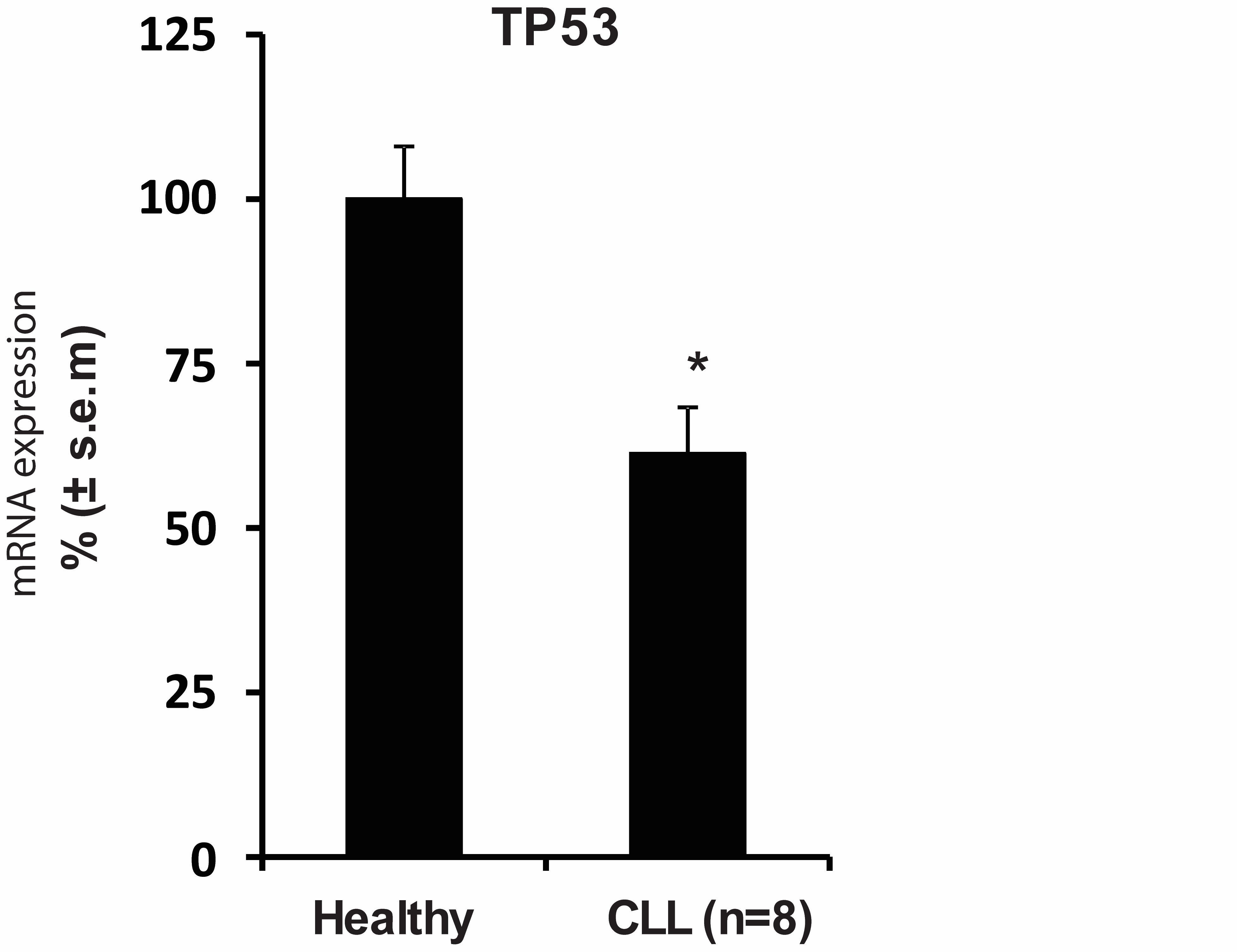


Healthy

n=3

CLL

n=8

**Supplementary Figure S1. CLL cells showed reduced TP53 mRNA expression levels.** Quantitative real time PCR (qRT-PCR) and unpaired t-test demonstrated significantly reduced TP53 mRNA expression levels (p ≤ 0.05) in CLL (n= 8) versus healthy (n=3) donor samples.

**Suppl. Figure S2**

**

**

**Supplementary Figure S2. TP53 activation by UV treatment and siRNA mediated knockdown of TP53 in healthy donor CD19+ cells.** Immunofluorescence of TP53 (green), CDKN1A (red) and DAPI (blue) in healthy donor CD19+ cells transfected with scrambled control siRNA (upper panel), scrambled control siRNA treated with UV induction of DNA damage activating TP53 (middle panel) and siTP53 (lower panel). Scale bar: 10 µm.

**Suppl. Table S1**

**

**

**Supplementary Table S1. Clinical information.** Table lists gender, age, binet-stage, treatment, chromosomal aberration as well as percentage of doublets detected in the CLL samples used in this study.

**Suppl. Table S2**

**Supplementary Table S2. Fold change of differentially expressed genes.** Specific values correspond to the heatmap shown in Figure 3A. Gene list and values have been obtained from a publically available RNAseq data set published by Ferreira *et al.* (2)

**Supplementary Material and Methods**

**Isolation of CD19+ cells**

Blood was taken from patients diagnosed with CLL (Rikshospitalet, Oslo University Hospital) and healthy volunteers (Ulleval University Hospital Blood Center). CLL patients were diagnosed according to standard morphologic, immunophenotypic and clinical criteria (1). The present study included a total of 20 patient samples. 17 out of 20 samples were derived from CLL patients who never received treatment; three samples were isolated from patients who receive treatment. Samples were obtained following informed consent using protocols approved by the Regional Medical and Health Research Ethics Committee of South-East Norway.

**Immunostaining of CD19+ cells**

Cells were fixed for 30 min in 3 % paraformaldehyde (Sigma, Missoury, USA), permeabilized for 15 min with 0.1 % TX100 (Sigma), and blocked for 15 minutes in PBS with 0.01 % saponin (Sigma) and 3 % (essentially fatty acid free) BSA (Sigma). Primary antibodies (Lamin B1 (Goat) (1:200; Santa Cruz, Texas, USA), Plk1 (Rb) (1:100; Abcam, Cambridge, UK), NuMA (Rb) (1:100; Abcam), AurB (Mo) (1:100; Abcam), Tubulin (Mo) (1:400; Sigma), and Actin (goat) (1:200; Santa Cruz)) were prepared in PBST with 3 % (essentially fatty acid free) BSA and 0.01 % Saponin and incubated over night at room temperature (RT). The next morning, coverslips were washed 3 x in PBST containing 3 % BSA and 0.1 % saponin before incubation in secondary Alexa fluor 488 Donkey anti-Mouse (1:500) (Invitrogen Massachusetts, USA), Alexa fluor 546 Donkey anti-Rabbit (1:500) (Invitrogen) and/or Alexa fluor 488 anti-Rabbit (1:500) (Invitrogen) or Alexa fluor 546 Donkey anti-Goat (1:500) for 3-5 hours. Coverslips were washed 3x 5 min in PBS with 0.01 % Saponin, rinsed with MQ H2O and mounted with Fluoromount G before imaging was performed using a 60x/1,4 DIC oil immersion objective on a LSM510 META confocal microscope (Zeiss, Germany). Imaging was performed using the Zen 2009 Blue software, followed by image processing in Photoshop. Files were exported as 16-bit RGB tiff files and placed into Adobe Illustrator to assemble the final figures. No image manipulation was performed, except mild adjustments of levels in Photoshop and cropping to appropriate size for final figures.

**Transient siRNA-mediated TP53 knockdown**

Delivery of siRNA was performed using the ECM 630 electroporator (BTX Harvard Apparatus), 250 mV, 960 μF, and 200 Ω. 50 nM siTP53 were transfected right after cell isolation (0h), and cells were cultured on inactivated CD40L feeder cells. After 48 hours, cells were harvested for immunofluorescence analysis. Knockdown efficiency of TP53 was between 70-80 %.

**References:**

1. Ferrante A, Thong YH: **Optimal conditions for simultaneous purification of mononuclear and polymorphonuclear leucocytes from human blood by the Hypaque-Ficoll method.** *J Immunol Methods* 1980, **36:**109-117.
2. Ferreira PG, Jares P, Rico D, Gomez-Lopez G, Martinez-Trillos A, Villamor N, Ecker S, Gonzalez-Perez A, Knowles DG, Monlong J, et al: **Transcriptome characterization by RNA sequencing identifies a major molecular and clinical subdivision in chronic lymphocytic leukemia.** *Genome Res* 2014, **24:**212-226.
